# The graph construction competency model for biology (GCCM-Bio): A framework for instruction and assessment of graph construction

**DOI:** 10.1093/biosci/biaf060

**Published:** 2025-06-20

**Authors:** Joel K Abraham, Elizabeth Suazo-Flores, Anupriya Karippadath, Alec Lamond, Susan Maruca, Eli Meir, Stephanie M Gardner

**Affiliations:** Department of Biological Science, California State University, Fullerton, Fullerton, California, United States; Department of Teaching, Leadership, and Professional Practice, University of North Dakota, Grand Forks, North Dakota, United States; Vanderbilt University, Nashville, Tennessee, United States; Department of Biological Science, California State University, Fullerton, Fullerton, California, United States; SimBiotic Software, Missoula, Montana, United States; SimBiotic Software, Missoula, Montana, United States; Department of Biological Sciences, Purdue University, West Lafayette, Indiana, United States; SimBiotic Software

**Keywords:** education, assessments, statistics, graph construction

## Abstract

Biologists represent data in visual forms, such as graphs, to aid data analysis and communication. However, students struggle to construct effective graphs. Although some studies explore these difficulties, we lack a comprehensive framework of the knowledge and skills needed to construct graphs in biology. In the present article, we describe the development of the Graph Construction Competency Model for Biology (GCCM-Bio), a framework of the components and activities associated with graph construction. We identified four broad knowledge areas for graph construction in biology: data selection, data exploration, graph assembly, and graph reflection. Under each area, we identified activities undertaken when constructing graphs of biological data and refined the GCCM-Bio through focus groups with experts in biology and statistics education. We also ran a scoping literature review to verify that these activities were represented in the graphing literature. The GCCM-Bio could support instructors, curriculum developers, and researchers when designing instruction and assessment of biology graph construction.

Calls for reforms to biology education across all levels encourage students to participate actively in science practices (AAAS 2011) to foster better scientific understanding. For instance, core competencies in the *Vision and Change* report, such as quantitative reasoning and communication with other disciplines, are essential for engaging in science practices (AAAS 2011). Facility with visualizations, such as graphs, is a critical component of quantitative reasoning and scientific literacy (Bray Speth et al. 2010) and, in the context of undergraduate biology education, has been included in the skills elaborated from the *Vision and Change* report (Clemmons et al. 2020). Graphs are one of the primary ways scientific data and ideas are communicated to science practitioners and the public (Mayes et al. 2014). As instruction expands beyond science content to the processes and practices of science, one can expect the importance of competence in reading and building graphs to increase for students.

Students at all levels regularly struggle with graphing (e.g., Bowen and Roth 2005, Metz 2008, Bray Speth et al. 2010, Glazer 2011). As is true with other scientific practices, student difficulties with graphing may be both a reflection and cause of broader difficulty in using data (Friel and Bright 1996, Bengtsson and Ottosson 2006, Harsh and Schmitt-Harsh 2016, Kjelvik and Schultheis 2019). Challenges with graph interpretation are more regularly discussed in the literature, but student challenges in graph construction are increasingly being studied (Angra and Gardner 2017, Gardner et al. 2021, 2024). Although undergraduate students may be skilled in basic aspects of graph construction, such as plotting data points (Padilla et al. 1986), they are challenged by other elements, such as selecting an appropriate graph type or identifying relevant variables (Bowen and Roth 2005, Angra and Gardner 2017, Gardner et al. 2021, Meir et al. 2023).

These difficulties may be partly due to the importance of disciplinary knowledge (i.e., knowledge about the nature of the graphed variables and the approaches or measurements used to acquire them) in graph construction (Bowen et al. 1999, Pfannkuch et al. 2004, Roth and Bowen 2001). For experts, graphing is a practice that is often habitual and is based not just on their ability to plot points but also on their knowledge of the system under study, data analysis and statistics, and the research question they are addressing (Roth and McGinn 1997). Novice graph constructors in biology are still developing disciplinary knowledge, which complicates their decisions, for instance, about whether data should be summarized or presented raw or how data should be arranged in a clustered bar graph (Angra and Gardner 2017). Students, particularly those earlier in their academic careers, are still developing an understanding of the complexity of data, which affects decisions about data presentation (Kjelvik and Shultheis 2019). Effective graphs communicate information in ways that the graph builder desires and in a manner that is interpretable by the intended audience; this requires reflection, with experts regularly making multiple graphs of the same data to determine which is most effective (Angra and Gardner 2017, Friel et al. 2001). Different aesthetic or structural choices affect how graphs are processed and interpreted (Tufte 1983). Therefore, even when students correctly plot data on appropriate graphs, decisions about such aspects as scaling, arrangement, and the use of colors or markers often vary and may not align with the intended purpose of the graph. Novices and experts differ in approach when graphing biological data (Angra and Gardner 2017). However, even practicing scientists struggle with graph construction (Diong et al. 2018); indeed, several recent articles in biology journals have offered guidelines for scientists on graph construction to improve communication about data in manuscripts (e.g., Weissgerber et al. 2015, Diong et al. 2018).

Some difficulties with graph construction may be exacerbated by inconsistent or limited instruction. Most instructors likely learned to graph through an apprenticeship model (e.g., as part of a research group). Therefore, they may not be as conscious of the specific knowledge or behaviors necessary for a given graphing task (e.g., Angra and Gardner 2016). Instructors do not regularly use class time to engage and enculturate students into the norms and behaviors of experts in graph construction and interpretation (Bowen and Roth 1998). Furthermore, instructors rarely enrich students’ experience with data and graphs, tend to use oversimplified graphs, and fail to deconstruct and analyze figures with students (Bowen et al. 1999). Similarly, research and assessment of student graphing are challenging to design without a clear understanding of the cognitive behaviors associated with competency in graph construction (e.g., Berg and Boote 2017).

We believe that a clear framework could support the development of more effective instructional and assessment tools for graphing in biology. Although disciplinary guidelines for what constitutes competence in graph construction are spread throughout the literature, a consolidated framework could provide structure to instructional or assessment planning for graph construction or guide targets for research on student competence in graphing. Other researchers have published assessment tools and instructional approaches for graph construction in biology (e.g., Webber et al. 2014, Angra and Gardner 2016, Harsh and Schmitt-Harsh 2016, Kjelvik and Schultheis 2019, Gardner et al. 2022, Harsh et al. 2023, Gardner et al. 2024); indeed, these papers informed or are based our own work. However, we saw a need to support researchers and instructors by providing a set of specific, assessable activities to guide them in supporting undergraduate biology students in their development of graph construction competency. In this article, we describe the development of a framework for graphing competency in biology, the Graph Construction Competency Model for Biology (GCCM-Bio). We believe the GCCM-Bio can be a tool to support researchers and instructors in designing assessment and instructional tools for graph construction for undergraduate biology students (table 1).

**Table 1.** Graph Construction Competency Model for Biology (GCCM-Bio) components, activity codes, and activity statements, with explanations and examples.

Component	Activity code	Activity statement
Data selection	Data type	Differentiates between quantitative (i.e., ratio, interval) and qualitative (i.e., ordinal, nominal) data
	Variable relevance	Selects variables for the graph that are relevant to a scientific claim in the context of a given research question, hypothesis, prediction, or objective
	Variable categorization	Identifies variables as related or causally linked in the context of a stated research question, hypothesis, prediction, or objective
	Data filtering or prioritizing	Plots appropriate data points, and appropriately excludes data points (e.g., missing data, corrupted samples), from each variable on the basis of data characteristics
Data exploration	Data form	Differentiates between data as a set of individual values (i.e., sample data) versus data as a distribution that could be summarized
	Data summarization	Plots individual or summarized data to communicate information efficiently for a given data set and intended purpose
	Statistics selection	If summarizing data, selects appropriate descriptive statistic for a given data set and intended purpose
	Data variability	Displays variation in data in a form appropriate for a given graph type and intended purpose
Graph assembly	Graph type	Selects a graph appropriate for the data type and intended purpose
	Data plotting	Plots data in the correct coordinates
	Graph structure	Follows disciplinary conventions in scaling, assignment, and orientation of graph axes and elements
	Graph labeling	Includes succinct axis labels and graph title or caption that effectively communicate the data plotted, in what way data have been transformed, and what the chosen graph elements (e.g., error bars, symbols, colors) represent
	Graph communication	Designs graph to efficiently communicate data and achieve aesthetic goals for a given purpose
Graph reflection	Data points	Extracts values of the sample or summarized data points from the graph
	Data description	Describes the characteristics (e.g., central tendency, variability) and patterns of the graphed data for the plotted values and graph type
	Graph selection	When one or more graphs are constructed, evaluates the affordances and limitations of each graph for exploring data characteristics or for supporting a scientific claim
	Scientific claim	Interprets the constructed graph to support a scientific claim in the context of a given research question, hypothesis, prediction, or objective

## Theoretical frameworks and context

Three theoretical frameworks guided us in developing the GCCM-Bio framework: metarepresentational competence, situated cognition, and evidence-centered design. A graph creator must have representational competence: knowledge of various representations’ identities, functions, and purposes (for a review, see Daniel et al. 2018). The conceptual framework of metarepresentational competence extends one step further to include an explicit reflective component regarding the appropriateness, quality, function, affordances, and limitations of one's own representations (diSessa 2004). Expert-like behavior is implicit in the metarepresentational competence framework, which provides a useful structure for understanding areas of competence with graphs. Another conceptual framework we made use of is situated cognition theory (Roth and Jornet 2013), which suggests that competencies around a practice, such as graph construction, differ across disciplines, and, therefore, instruction in that practice should be explicitly embedded in its disciplinary context (Roth and McGinn 1997). What is considered an appropriate graph type (e.g., a bar graph of categorical and continuous data) or element (e.g., standard error bars) or salient interpretations of global patterns is influenced by the culture or nature of a given academic discipline or audience. Finally, the development of the GCCM-Bio is informed by the evidence-centered design assessment framework (Mislevy et al. 2003). Evidence-centered design is a structured method for constructing complex assessment instruments (Mislevy and Haertel 2006). One element of the evidence-centered design conceptual assessment framework is the student model, which specifies the knowledge an individual must have or the actions that an individual must take to be competent in performing some task at the desired level. We developed the GCCM-Bio to serve as the student model for our broader effort to develop and validate performance-based assessments and instructional tools for graph construction in biology; for a fuller discussion of the evidence-centered design approach in this effort, see Meir and colleagues (2023).

The GCCM-Bio is situated in the context of biological science and only includes graph interpretation as a component of graph construction. We recognize that scientists in every discipline make abundant use of graphs to explore, analyze, and communicate data, hypotheses, and concepts in teaching and research. However, we limit the scope of this framework to biology because the approach to graphing, graph conventions, and the language used to describe the activities and knowledge around graph construction differ across scientific disciplines. Even within biology, the type of data graphed in, the assumptions in the research design of, and the scientific claims made with graphs can vary, and, therefore, expertise in graph construction in one area of biology does not necessarily translate to other areas (Roth and Bowen 2001). Focusing on biology allows us to avoid some of the translation problems that are bound to happen when working across disciplines (Rabin et al. 2021). However, graph construction naturally intertwines with constructs of data literacy (Mandinach and Gummer 2013), quantitative literacy (Mayes et al. 2013), and statistical literacy (Gal 2004, Sharma 2017); they are all related to how we make sense of and communicate ideas about data. Accordingly, perspectives from literature and experts in those areas are also crucial to support the development and help ensure the relevance of the GCCM-Bio. These constructs are relevant to any discipline in which data are used. Therefore, although we place boundaries on this work, we believe the GCCM-Bio can serve as a useful starting point for future discussions about how graph construction competence is operationalized across other academic disciplines. Below, we first present the final form of the GCCM-Bio. We then describe the process we took to develop this framework, followed by a brief discussion of potential use cases for it.

## The GCCM-Bio

The GCCM-Bio incorporates knowledge and research from our research team, published literature on graphing practices and literacy, and focus group participants with independent and broad perspectives. We believe it offers a novel and solid foundation for understanding and planning instruction, assessment, and research on graph construction in biology. We broke the graph construction process down into four components, the broader conceptual categories of graphing competencies. Nested within each component are four to five activities, the specific cognitive or physical actions that a biologist would undertake while constructing a graph (table 1; expanded descriptions and examples appear in supplemental table S1). For example, the data plotting activity (plotting data points on a graph) is part of the graph assembly component. When referring to both components and activities, we use the term *elements*. As much as possible, we endeavored to make all the activities independent, meaning someone could show expert-like competence in any activity while showing novice-like challenges in any other. Collectively, with practice and learning across these activities, one could become competent with graph construction in biology.

The GCCM-Bio includes the range of activities that could occur during graphing, but not all are necessary to complete a given graph. For example, when constructing a graph of biological data, one must complete activities under the graph assembly component, such as selecting the type of graph (the graph type activity) or plotting data onto the graph (the data plotting activity). However, graph selection is only relevant if one considers or creates multiple graphs, which is needed to reflect on the affordances and limitations of different graph types. Some activities are relatively discrete (e.g., data plotting), whereas others are synthetic or necessarily co-occur with other activities (e.g., graph selection, graph communication). We sought to communicate what activities someone competent in the broader practice of graph construction would do; no scales or levels of expertise are included. We believe that instructors or researchers can best determine what constitutes expertise for a given population or scenario but that these activities provide a framework for constructing learning objectives to guide instruction and assessment.

## The development of the GCCM-Bio

We developed the GCCM-Bio across three stages. In stage 1, we constructed a prototype framework using our research and teaching experiences, knowledge of published literature, and discussions within our research group and with our advisory board. In stage 2, we sought to improve on the GCCM-Bio by asking for critical feedback from a series of focus groups with experts in biology education, statistics education, and quantitative thinking, gathering support for its relevance and utility. In stage 3, we conducted a scoping literature review to verify that the components of our framework are represented in the graphing literature. Below, we detail each of these stages of development.

### Stage 1: Research group discussions

We started the development of our first draft of the GCCM-Bio on the basis of our collective experiences. We reflected on our own graphing process and approaches to instructing students in graph construction. JKA, SMG, ES-F, and EM had experience designing assessments and research on undergraduate student graph construction competence. Still, we heavily relied on the work of Angra and Gardner (2016) and our observations of students and biology faculty constructing graphs in research interviews conducted as part of our development of a performance-based graphing assessment (Meir et al. 2023) to focus our discussion. Three authors (JKA, EM, ES-F) constructed a prototype of the GCCM-Bio and shared it with the research group for a round of comments and revisions. At this time, we agreed on a structure (described in detail above) that was inspired, in part, by work done in the ACE-BIO Research Coordination Network (Pelaez et al. 2016, 2022).

### Stage 2: Expert focus groups

After completing the prototype GCCM-Bio, we recruited experts to participate in focus groups to discuss its strengths and weaknesses with respect to scope, structure, content, and utility. We chose potential participants on the basis of their relevant expertise in areas related to graphing in biology; specifically, we sought participants with expertise as introductory biology instructors, biology education researchers, or statistics or quantitative education researchers. We gauged their expertise on the basis of their publication history, participation in NSF Research Coordination Networks, and attendance at relevant conferences. We excluded anyone from the potential participant pool with whom we had collaborated on publications, grants, or advisory boards. After collating a list of 20 experts, we emailed them to invite them to join an online focus group to comment on the revised GCCM-Bio; of the 20, 8 agreed to participate. The eight participants spanned a range of professional levels, research areas, and institution types (table 2). The participants first responded to a brief online survey to schedule their focus groups and to receive a copy of the GCCM-Bio before the meetings. We attempted to match research interests, but we scheduled the participants for one of three focus group sessions on the basis of their availability.

**Table 2.** A description of our focus group participants. Some details about participants’ professional positions, institutions, and research areas were modified to preserve confidentiality.

ID	Position	Institution	Research Area	Teaching
FG1	Instructor	DU	Data science education, quantitative thinking	IM, INM, UD
FG2	Midcareer professor	DU	Visualizations, genetics, molecular biology education	IM, UD
FG3	Researcher	DU	Quantitative thinking, data literacy, visualizations	INM
FG4	Early-career professor	DU	Visualizations, undergraduate research experiences	IM, INM
FG5	Researcher	RS	Quantitative thinking, data literacy, visualizations	–
FG6	Early-career professor	DU	Quantitative thinking, undergraduate research experiences	IM, UD
FG7	Late-career professor	MU	Statistics education	ST
FG8	Early-career professor	DU	Statistics or data science education	ST, DV

*Abbreviations:* DU, doctoral granting university; DV, data visualization; IM, introductory biology for majors; INM, introductory biology for nonmajors; MU, master's granting university; RS, research station; ST, statistics; UD, upper-division biology.

The focus group protocol was approved by the California State University, Fullerton, institutional review board (human subjects research project no. 18–19-505). The focus groups followed a semistructured format, with defined questions and follow-up probing questions driven by the participants’ responses. After introductions and a project description, we asked the participants to discuss the four questions listed below. At the time, we had not yet decided on a name for our student model; we've added the name to the questions to improve clarity, but the text is otherwise identical to what the participants received: “To what degree does the [GCCM-Bio] capture important skills for graph construction in the context of biological *experimentation*?” (*Experimentation* was revised to *research* in the final focus group on the basis of feedback in first two groups.) “What skills related to graph construction do you think the [GCCM-Bio] is missing?” “Consider the [GCCM-Bio] as a whole: If you had data on the graph construction skills from this model for your students, how well would you feel those data would, or would not, represent the graph construction ability that you care about?” and “Do you have anything else to add about the [GCCM-Bio], or about our project in general?”

Author SM, who did not participate in the design of the GCCM-Bio beyond editorial comments, moderated the focus groups. Authors ES-F and JKA took notes, managed the sessions, and recorded the conversations. Authors JKA, AL, and ES-F transcribed and summarized the participants’ comments. The research group discussed the summaries of the focus groups, identified valuable feedback for improving the GCCM-Bio, and integrated those changes into the framework.

The focus group participants responded positively to the GCCM-Bio and its usefulness as a tool for instructors and researchers. They agreed that the GCCM-Bio captures important activities central to graphing biological data and aligned well with previous work in quantitative thinking, data literacy, and statistics education (supplemental table S2). The primary value of the GCCM-Bio was seen in the areas of instructional design and research. The participants regularly noted how the GCCM-Bio could help instructors focus on fundamental skills within graphing, highlight potential gaps in instruction, and provide the foundation for new assessment tools, such as rubrics, to be used in research and teaching (supplementary table S2).

The most substantial changes to the GCCM-Bio were made on the basis of the focus groups’ comments about the language and content of the fourth component (supplemental table S2). The early version of GCCM-Bio titled this component *graph interpretation*, but our participants felt that this title was vague in focus and inclusive of things outside of graph construction. We revised the title of the fourth component to *graph reflection*, which better captures interpretation as an evaluation process for the graphs one builds. We also changed the name of the activity code from *draw conclusion* to *scientific claim* and edited the associated activity statement. Several of the focus group participants rightly pointed out that our description of graph construction should include instances when it is premature to draw conclusions; therefore, language about drawing conclusions might reinforce misconceptions in students that final answers are typical in biology. Criticism by several of the participants in the focus group led us to expand the title of the graph trend activity to the broader *data description*, which better captures what biologists might consider when evaluating their graphs. Finally, we added the graph selection activity to the graph reflection component (table 1). Graph selection was recommended in the focus groups as a critical aspect of reflection, describing the often iterative and reflective critical practice of graphing by experts in biology (diSessa 2004). This task was an important part of the step-by-step guide to graph construction described in Angra and Gardner (2016), so it was also consistent with our previous work.

We incorporated minor editorial recommendations from the focus group on other activities (data type, data summarization) and changed the phrasing that suggested that research in biology is limited to experimentation. More broadly, the focus group participants recommended ways to better communicate potential uses of the GCCM-BIO and to provide clarifying statements for each activity when sharing the GCCM-BIO with faculty to support its use; those suggestions are incorporated into this article.

### Stage 3: Scoping literature review

We next conducted a scoping literature review (Grant and Booth 2009, Munn et al. 2018) to ensure that the activities in the GCCM-Bio were represented in graphing publications. Scoping literature reviews differ from systematic literature reviews in that they are generally broader in scope and do not provide a summary and critique of the papers’ findings; instead, they can provide insight into how concepts are used in the literature and can add clarity in definitions (Grant and Booth 2009). In this case, the scoping literature view provided us with a better understanding of the nature of graphing competence in the literature, which assisted us in further refining our framework and confirming that it includes relevant components and activities.

We began our search in early 2019; ES-F and SMG developed the search protocol, which was presented to the research team and approved by all. ES-F searched for articles in two databases: Scopus (limited to the education field) and EBSCO. Our selection of the databases was based on previous use of those databases to identify relevant articles for graph construction. The following keywords were used for the search: *graph* OR *graphs* OR *graphing* in paper titles AND interpret* OR construct* in paper abstracts. The search was limited to peer-reviewed journal articles written in English and those in the education field in the Scopus database. This process led to 167 articles in EBSCO and 359 articles in Scopus. In consultation with SMG, ES-F first read the abstracts for each paper and eliminated those not explicitly educational or from journals judged by the research team to be predatory; this latter designation was an informal judgment based solely on the authors’ experience. After merging the results from both searches and eliminating replicates, they ended with 107 articles. Some of the articles were already known to the authors, and the list included the authors’ own work, which increased confidence that the search was targeting relevant articles. JKA, SMG, and AK cocreated and piloted an approach to independently code the articles.

JKA repeated the search independently in late 2020 and found 85 articles that were published after or that were left out of the initial pool of articles coded. JKA reviewed the abstracts of each of the articles and removed 43 on the basis of the guidelines described above, yielding 42 additional articles for coding. This yielded a total of 149 articles in the scoping review to identify which, if any, aspects of the GCCM-Bio were present (see supplemental tables S3 and S4).

SMG and JKA read and coded a shared subset (*n* = 36, 23%) of the 149 articles, stratified to ensure a similar percentage of articles across both search periods. They calculated intercoder agreement and Cohen's kappa for each GCCM-Bio activity across that subset (table 3). The percentage agreement ranged from 69%–97% across the activities. On the basis of the guidelines suggested by Landis and Koch (1977), which are commonly used by researchers in categorizing Cohen's kappa values, the range of kappa values (.42–.87) for 12 of 17 GCCM-Bio activities fell under the moderate to near-perfect agreement categories. Three activities (variable categorization, data variability, and graph structure) fell under the fair category, with kappa values of .36–.38, whereas the values for data filtering or prioritization and data form fell under slight or poor agreement (–.04–.12). We recognize that, for assessment purposes, those low values would preclude the use of a tool without further revision. However, we felt comfortable proceeding in this case because our intent was to corroborate the presence of activities across a body of diverse publications rather than to assess or diagnose individual publications. For the remaining articles, either JKA or SMG coded for the activities alone.

**Table 3.** Representation of GCCM-Bio activities in publications from scoping review and intercoder reliability.

GCCM component	GCCM activity	Percentage of the total papers presented (*n* = 149)	Percentage agreement (*n* = 36)	Cohen's kappa (*n* = 36)
Data selection	Data type	12	92	.72
	Variable relevance	11	92	.48
	Variable categorization	22	81	.36
	Data filtering or prioritization	5	89	–.04
Data exploration	Data form	11	78	.12
	Data summarization	13	89	.44
	Statistics selection	11	92	.36
	Data variability	11	97	.87
Graph assembly	Graph type	28	78	.54
	Data plotting	42	78	.56
	Graph structure	42	69	.38
	Graph labeling	35	78	.56
	Graph communication	30	78	.55
Graph reflection	Data points	62	86	.62
	Data description	77	86	.53
	Graph selection	16	78	.48
	Scientific claim	34	89	.75

*Note:* The paper references from the scoping review for each activity can be found in supplemental table S3.

This scoping literature review provided additional, if less direct, support for the contents of the GCCM-Bio as important elements of graph construction. We found examples of all GCCM-BIO activities represented in these articles, with the highest representation in the graph assembly and graph reflection components (table 3). For example, data description and data points were found in over half of the articles. The high frequency of activities from our graph reflection component likely stems from its overlap with graph interpretation, a more commonly studied science practice. Other activities were seen less frequently (in approximately 10% of the articles), such as variable relevance and statistics selection.

Given our aim of corroborating the importance of the GCCM-Bio elements rather than completing a systematic review of the literature, we were comfortable using data from a single coder (JKA or SMG) across the complete set of articles. However, we acknowledge several important limitations in our approach that argue for caution in drawing general inferences from the results of the scoping review. First, our ability to detect the presence of the different activities likely varied. For instance, if only a sample of items from a graphing assessment was published, we were only able to code activities on the basis of those items or clear descriptions in the text. The low kappa values for several activities also reduced confidence that we were as consistent in capturing them in our review. The purpose of the articles was different from how we use the GCCM-Bio elements in our own work, and some activities related to structural components of graph construction (e.g., data plotting) may be easier to identify consistently in articles than more abstract activities (e.g., variable relevance, data filtering or prioritization). Finally, the patterns we found were almost certainly influenced by our search terms; the inclusion of more articles related to data literacy, for instance, might have yielded more instances of activities under the data description or data exploration categories.

## Potential of the GCCM-Bio in supporting graph literacy in biology

The evidence we collected suggests that the elements of the GCCM-BIO align very well with the perspectives of experts in biology education, data visualization, quantitative literacy, and statistics and are also represented in the literature on graphing across fields. Therefore, we have confidence that the current version of the GCCM-Bio is an important resource to support instructors in designing instructional and assessment tools for graphing in undergraduate biology students, researchers in their ability to broadly assess graph construction competency, and students for self-reflecting on their own graphing process. The GCCM-Bio has already been used to support the design of new performance-based assessments of graph construction competence (Meir et al. 2023), and we believe we and others will develop other new specific use cases for the GCCM-Bio in the future.

### Supporting graph construction competence

A recent essay summarizing the literature on designing effective instruction of graphing competence (Gardner et al. 2024) recommends the following: Use data that engage students. Teach graphing grounded in the discipline. Practice explicit instruction. Use real-world messy data. Use collaborative work. Emphasize reflection.

The GCCM-Bio provides the foundation for supporting the explicit instruction and grounding that instruction in the discipline that is called for in this essay. Below, we briefly describe how two of the authors (JKA and SMG) each make use of this framework to transform their own teaching.

## Upper-division lecture and lab biology course

JKA found immediate value in the GCCM-Bio as part of an ongoing redesign of his upper-division course-based undergraduate research experience (CURE). This course relies heavily on the use of primary literature, so JKA regularly asks students to interpret graphs. However, he also tasks students to generate conceptual or data graphs as part of oral and written assignments and during exams, and at the end of the course, the students submit graphs as part of their research papers and presentations on their group and independent projects. Therefore, their performance in the course is based in part on their graph construction skills.

**Figure 1.**
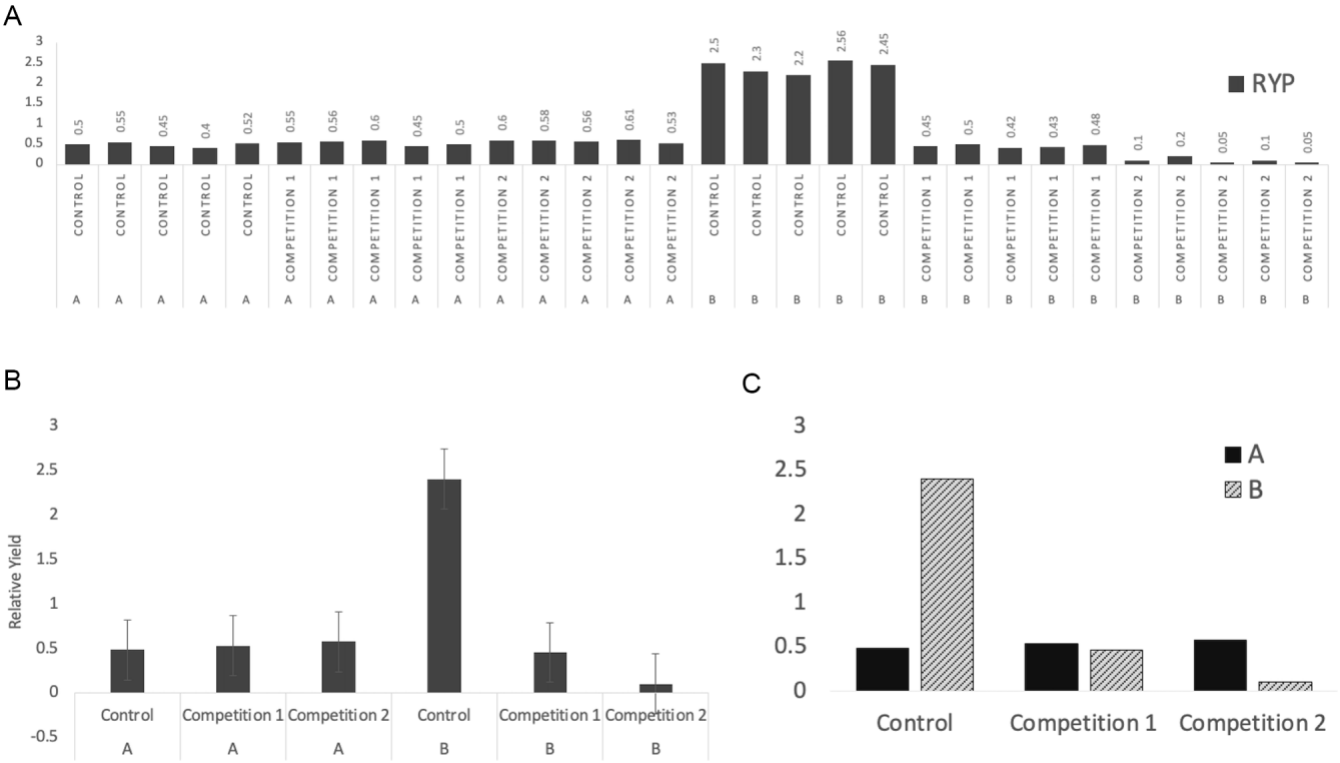
Three example graphs designed by Abraham that represent a mosaic of choices commonly made by students when constructing graphs for an upper-division plant ecology course-based undergraduate research experience. The graphs are structured on the basis of common student research project designs, which often compare the influence of a stimulus (1 versus 2) on the competitive outcomes (relative yield per plant) across two species (A and B). These graphs show a mix of appropriate and inappropriate steps taken across activities in the GCCM-Bio.

JKA noticed that students regularly struggled with making informative or effective graphs to communicate their thoughts or results. This was particularly apparent in the research papers and presentations produced at the end of the CURE. As a visual aid, the recreated graphs shown in figure 1 communicate a mosaic of some of the most common problems JKA observed in the students’ bar graphs. For instance, the students in the course regularly plotted individual (raw) data in cases where summarized data would be more appropriate and would allow for comparisons across groups (data summarization). The students commonly arranged the columns in ways that reduced a reader's ability to discern differences across the relevant groups (graph communication). The indicators of data variability were rarely included, and the students rarely considered other graph types, such as categorical scatterplots, that would show more aspects of the data distribution (data variability, graph type, graph selection). Students may use the suggested labels, colors, or legends in popular graphing software without considering whether they are necessary or support graph interpretation (graph labeling).

JKA began a redesign effort while the present authors were completing the development of the GCCM; this redesign is still ongoing and will be implemented in the next iteration of the course. Using the GCCM-Bio, JKA reflected on which elements were well integrated, touched on, or ignored in the course. JKA also reviewed the students’ final presentations, research papers, and instructional materials from his previous three classes. As an example, the instructions provided to the students in two different lab worksheets were as follows: “Construct 1–2 conceptual graphs that show what the data would look like if your hypothesis were supported” and “Include the graph(s) (with all required elements) that your group feels would best communicate the results of your experiment. Remember to include figure captions for each graph.”

Although the course already relied heavily on collaborative analysis of real-world data generated by students, JKA identified several areas for growth in his approach to instruction that the GCCM-BIO directly supports. The most obvious element lacking was explicit instruction, including clear learning objectives, in important areas of graph construction. In particular, JKA noted the poor course coverage of graphing activities under data selection, vague descriptions of graphing tasks, and inconsistent opportunities for the students to create and reflect on multiple graphs.

The GCCM-Bio provides the language and framework for instructors to model their own graphing practices for students or to develop learning objectives for assignments to strengthen student skills. For instance, JKA is revising the course labs, which have historically been used to teach specific biology research skills and concepts, to include learning objectives for graph construction developed from the GCCM-Bio. Examples of draft learning objectives from one revised lab in which students will create graphs of student-generated data include the students being able to select an appropriate graph type for the type of data being visualized (graph type) and to justify the selected graph variables on the basis of the predictions of the study (variable relevance).

Although any graphing exercise could prompt introspection in students, JKA’s graphing tasks are missed opportunities to emphasize reflection and ensure the graphs are grounded in the discipline. Although his students regularly interpret various graphs in the primary literature, the course assignments provided practice only in basic line, bar, and scatterplots. As part of the scaffolding for the final group research presentations and papers, JKA is developing new course exercises for use toward the end of the course, in which pairs of students must generate and contrast two different graphs of the same data using AI tools (graph reflection). This approach will provide multiple opportunities for practice while removing the time and expertise constraints associated with generating more complex graphs through traditional software. This practice will better support student development of graph construction competence, which should lead to higher-quality graphs in the final research project.

## Intermediate-level physiology lab course

This course taught by SMG similarly combines the GCCM-Bio and graph construction framework (Gardner et al. 2024). The course and previous instructional interventions have been described elsewhere (Gardner et al. 2022), but briefly, in this course, students work in teams to articulate hypotheses and predictions that follow from them (Karippadath et al. 2025) to guide experimental designs, data collection, data analysis and visualization, and making claims related to the prediction. Early in the semester (week 2), SMG engages in formative assessment and a guided discussion around data analysis and visualization on the basis of a simple data set that the students had gathered to understand what they know and know how to do while also helping them learn about course expectations, guides, and rubrics (Angra and Gardner 2016, 2018, Gardner et al. 2022). All GCCM-Bio elements are explicitly incorporated into this intervention and guided discussion.

Not included in Gardner and colleagues (2022) is an interactive portion initiated in the spring of 2021 that serves formative purposes and lays the foundation for practice throughout the semester. The activity is part of the larger guided discussion mentioned above and is based on figure 1 in Weissgerber and colleagues (2015), in which the students are asked which of the categorical scatter plots (also called *strip* or *bee swarm plots*) could represent the data plotted in a bar graph of averages with error bars. The categorical scatter plots show two conditions and the raw data from each condition and illustrate data sets of unequal sample size, bimodal groupings within each treatment, single outliers, and a normal distribution for each condition. To date, less than 20% of the students realize that any of the categorical scatter plots could represent the raw data summarized in the bar graph. This then allows SMG to discuss with the students the importance of exploring and representing raw data not only as a precursor to using descriptive statistics (e.g., means or medians) but also to communicate information about the data in a finalized graph. Furthermore, the importance of labeling graphs in such a way so that readers know what each element is (e.g., error bar type) and that more than one graph could be appropriate on the basis of the data, your purpose, and transparency in communication. She also shows them that descriptive statistics can be added (e.g., mean line or error bars) but also more elaborate representations such as incorporating box and whisker plots or the bar graph with error bars.

The GCCM-Bio elements in this guided discussion and formative assessment are data exploration (data form, data summarization, statistics selection, and data variability), graph assembly (graph type, Graph labeling, graph communication), and graph reflection (graph selection and graph communication).

This activity does not take long to incorporate into a lesson (about 15 minutes) but is rich, incorporating many GCCM-Bio elements and providing the students with a foundation from which to build throughout the semester, gaining practice and feedback along the way to develop competence.

## Conclusions

The practice of graph construction is universally recognized as central to biology. Graphs are one of the most powerful and common ways to communicate information about data, and the ability to construct effective graphs is recognized as a critical component of quantitative literacy. As important as graphing is for a complete education in biology, students and instructors are often frustrated by the challenge of teaching and learning graphing. The GCCM-Bio is a novel resource that summarizes the key elements of graph construction in biology; we hope other biologists will find it helpful in supporting teaching and research training in quantitative and visual literacy. Although we make it available now in its current form, we will continue to update it as the community provides additional feedback on the framework's structure, content, and use.

In addition, we plan to identify connection points between the GCCM-Bio and graph construction practices in other areas of science. There is still much to learn about how students approach graphing, what instructional approaches might improve graph competency, and which assessment formats and designs best illuminate competency levels. We look forward to joining others in developing new approaches to support effective instruction and novel research on student graphing competency.

## Supplementary Material

biaf060_Supplemental_File

## Author Biography

Joel K. Abraham (jkabraham@fullerton.edu) is a professor in the Department of Biological Science at California State University, Fullerton, in Fullerton, California, in the United States. Elizabeth Suazo-Flores is an assistant professor in the Department of Teaching, Leadership, and Professional Practice at the University of North Dakota, in Grand Forks, North Dakota, in the United States. Anupriya Karippadath is a STEM development specialist at Vanderbilt University, in Nashville, Tennessee, in the United States. Alec Lamond is enrolled in a master's degree program in the Department of Biological Science at California State University, Fullerton, in Fullerton, California, in the United States. Susan Maruca is the vice president and director of content development at SimBiotic Software, in Missoula, Montana, in the United States. Eli Meir is the past president and current director of research at SimBiotic Software, in Missoula, Montana, in the United States. Stephanie M. Gardner is an associate professor and associate head for curriculum, teaching, and learning in the Department of Biological Sciences at Purdue University, in West Lafayette, Indiana, in the United States. SimBiotic Software, which employs authors SM and EM, may eventually develop a product for sale that would be designed using the GCCM-Bio. No other authors have any conflicts of interest.
