# Supplementary material for: The graph construction competency model for biology (GCCM-Bio): A framework for instruction and assessment of graph construction
Source: Bioscience. 2025 Jun 20;75(8):649–58. doi: 10.1093/biosci/biaf060 (PMC12352301; doi:10.1093/biosci/biaf060)
Supplement: biaf060_Supplemental_File [file biaf060_supplemental_file.docx]

| Table A. Graph Construction Competency Model for Biology (GCCM-Bio) with explanations and examples. | | | |
| --- | --- | --- | --- |
| **Component** | **Activity Code** | **Activity Statement** | **Explanation and Context** |
| Data Selection | Data Type | Differentiates between quantitative (i.e., ratio, interval) and qualitative (i.e., ordinal, nominal) data types | Before graphing data, one must account for the characteristics of those data. For instance, simple scatter plots are designed to show relationships between two different continuous datasets, while bar graphs may show continuous data as it changes across discrete categories. The nuances of data type can influence graph selection and other decisions about data presentation. |
|  | Variable Relevance | Selects variables for the graph that are relevant to a scientific claim in the context of a given research question, hypothesis, prediction, or objective | The GCCM-Bio specifically focuses on graph construction in biology, and thus graphs that are meant to communicate ideas in biology. Every graph shows relationships among variables; the design of effective graphs requires the graph constructor to consider which of the many possible variables that could be graphed, should be graphed. The relevance of any variable is determined by the question or objective that requires a graphical representation of data. |
|  | Variable Categorization | Identifies variables as related or causally linked in the context of a stated research question, hypothesis, prediction, or objective | This activity is tightly linked to Variable Relevance but differs in that it is a recognition of the presumed relationship among the variables being graphed. This activity incorporates the theoretical models underlying the research as part of the graph-building process. This becomes particularly relevant in graphs with more than two variables included, as decisions must be made about how variables should be associated spatially within the graph. |
|  | Data Filtering/ Prioritizing | Plots appropriate data points, and appropriately excludes data points (e.g., missing data, corrupted samples), from each variable based on data characteristics | When graphing authentic data as a form of data analysis, decisions must often be made about the inclusion or exclusion of data. For instance, outliers may or may not alter one’s interpretation of patterns within a graph in ways that differ from their impact in statistical tests. |
| Data Exploration | Data Form | Differentiates between data as a set of individual values (i.e., sample data) versus data as a distribution that could be summarized | Before graphing, one must recognize that data can be presented as individual data points or in summarized form. Common data summarizations include central tendency (i.e., mean, median, mode) or data range. |
|  | Data Summarization | Plots individual or summarized data to communicate information efficiently for a given data set and intended purpose | Graphs designed to present general trends in data from different populations may sometimes be most efficient with summarized data, while at other times the distribution of data or the purpose of the graph may require that individual data points are included. |
|  | Statistics Selection | If summarizing data, selects appropriate descriptive statistic for a given data set and intended purpose | When summarizing data, it is important to note that some descriptive statistics may be more effective at conveying information about the data than others; in some instances, a statistic might be inappropriate (e.g., presenting the average of nominal data). |
|  | Data Variability | Displays variation in data in a form appropriate for a given graph type and intended purpose | When constructing a graph of summarized sample data, it is generally important to include an appropriate element, such as error bars on a bar graph, to communicate the variability in the data. However, if a graph shows raw data, elements like error bars should not be used. |
| Graph Assembly | Graph Type | Selects a graph appropriate for the data type and intended purpose | There are many different graph types, although students most frequently use and encounter line graphs, bar graphs, scatter plots, histograms, and pie charts. Each graph type is appropriate for given types and forms of data. Some are more efficient than others given a particular purpose or audience. |
|  | Data Plotting | Plots data in the correct coordinates | Plotting data is one of the earliest graph construction skills that students learn and is commonly automated when graphing of data is done using computer-based approaches. However, graphing by hand in classroom activities or on paper-based assessment tools often requires manual plotting. |
|  | Graph Structure | Follows disciplinary conventions in scaling, assignment, and orientation of graph axes and elements | Research labs, academic journals, or fields of biology may differ in their guidance or conventions when graphing data (e.g., inclusion of breaks in the y-axis, placing the dependent variable on the Y-axis), while other conventions are universal (e.g., setting a scale that allows a reader to properly interpret the most important information in the graph). |
|  | Graph Labeling | Includes succinct axis labels and graph title or caption that effectively communicate the data plotted, in what way data have been transformed, and what the chosen graph elements (e.g., error bars, symbols, colors) represent | Labels, titles, and captions provide the necessary contextual information to support communication of the results, aspects of the data, and which interpretations are most important. For instance, error bars commonly represent standard deviation, standard error, or 95% confidence intervals, all of which communicate different information about variation and the nature of relationships across groups. Captions must communicate such that someone with sufficient content knowledge about the topic can interpret the graph without the use of additional text. |
|  | Graph Communication | Designs graph to efficiently communicate data and achieve aesthetic goals for a given purpose | Graphs, primarily, are a tool to communicate information efficiently and effectively. During the design process, decisions must be made about such things as selecting which colors or markers to use, the “ink to information” ratio, or the font size on the axes. This is an example of a synthesis activity; it will typically be undertaken simultaneously with other activities. |
| Graph Reflection | Data Points | Extracts values of the sample or summarized data points from the graph | After building a graph, part of the reflection process includes a review of at least some of the data at the individual level. For example, one might check if the maximum and minimum values match expectations based on the data as a form of quality control. |
|  | Data Description | Describes the characteristics (e.g., central tendency, variability) and patterns of the graphed data for the plotted values and graph type | One reason graphs of data often replace data tables is that they more efficiently illustrate patterns in and characteristics of the data. For instance, a common recommendation after collecting data is to create quick plots and summary tables of data to look for patterns in its distribution, identify potential correlations, and identify outliers. |
|  | Graph Selection | When one or more graphs are constructed, evaluates the affordances and limitations of each graph for exploring data characteristics or for supporting a scientific claim | Experts often build multiple graphs of the same data and later decide which best communicates information for an audience and purpose. One complex graph may be best suited for exploring nuances of a finding at a conference with one’s peers, while a simpler graph might be necessary to highlight a key pattern when teaching introductory students. |
|  | Scientific Claim | Interprets the constructed graph to support a scientific claim in the context of a given research question, hypothesis, prediction, or objective | Graphs in science are tools for communicating both data and ideas. Every graph in science, whether illustrating a reasoned idea or patterns in data, is a statement about how relationships among things in the natural world are structured. Graph construction requires, as part of the reflective process, an evaluation of the graph(s) produced with regard to how well that statement (i.e., scientific claim) was captured by the graph. |

| Table 2: Representative examples of positive and constructive quotes about the GCCM-Bio prototype framework from Focus Group participants. Some details about participants' professional positions, institutions, and research areas were modified to preserve confidentiality. DU = Doctoral Granting University; MU = Master's Granting University; RS = Research Station; IM = Introductory Biology for Majors, INM = Introductory Biology for Non-Majors; UD = Upper-Division Biology, ST = Statistics, DV = Data Visualization. | | | |
| --- | --- | --- | --- |
| **ID** | **Research Area** | **Q1/Q3/Q4: Representative Positive Comments** | **Q2/Q4: Representative Critical Comments** |
| FG1 | Data Science Education, Quantitative Thinking | "It helps a faculty member potentially identify blind spots, right things that they were assuming that students would already sort of know about, which we all struggle with…once you've sort of articulated all of these potential skills, knowledge, bits and abilities, trying to identify task structures that would give students opportunities for practice and what kind of feedback you would give" | "In addition to title and statement, it might be helpful to have some operationalization of what those statements look like…sort of an inference about what students are able to do." |
| FG2 | Visualizations, Genetics/ Molecular Biology Education | "I liked the way that you did this, I have some really good ideas about how to think about making graphs and I liked the way that you broke it down…I think like each of these kind of signals to me things that I should be thinking about talking to my students about…all these things I maybe assumed that they knew" | "There's nothing in the model about using software to build graphs. And that is something that we kind of expect our students to do…Obviously, we want them to be use their knowledge to pick the right kind of graph using the concepts that you have in here. But, I wonder if you don't want to say something a little bit more explicit about selecting tools?" |
| FG3 | Quantitative Thinking, Data Literacy, Visualizations | "I saw this as being very useful for a rubric both for instructors and for research purposes…And I think that would be useful for instructors as well…I think the bones there are really good for being a rubric… especially this graph assembly part. I know some collaborators who put together a graphing rubric and it matches almost exactly to this. So, it's really good." | "The word conclusion kind of brings about misconceptions to me about the scientific process. So, I might change that to more in line with like, making a claim from the data. Because 'conclusion' can often leave students with the impression that after they do an inquiry project or an experiment that they're done, but in reality, most of the time after they've analyzed the data, there's a lot more questions." |

| **ID** | **Research Area** | **Q1/Q3/Q4: Representative Positive Comments** | **Q2/Q4: Representative Critical Comments** |
| --- | --- | --- | --- |
| FG4 | Visualizations, Undergraduate Research Experiences | "it would encourage faculty to actually look at and see what students are not capable of being able to do and identify problem areas in fundamental issues that they may have… a lot of the data or a lot of the categories that are listed here are things that align well with literature and things I try to follow along with a little bit in my class" | "It may be as simple as [changing it] to biological research or add something to that regard… in our class, we tried to not just discuss experimentation, but also observational types of research or even descriptive studies to some degree." |
| FG5 | Quantitative Thinking, Data Literacy, Visualizations | "Overall, it had the things that I would have expected, or the things that popped into my mind…I did like the focus on that when a student did graph something like an average that they were representing that within their axes labels." | "Teachers also tend to avoid the word conclusion…because you don't want to indicate that science is a concluded process…you can both look at a graph, do some interpretation of that graph, but it's a separate process to then use what you've interpreted out of the graph to construct an argument or construct an explanation." |
| FG6 | Quantitative Thinking, Undergraduate Research Experiences | "You have done a good job capturing a lot of the things that I think about and have actually gone much more in depth than I've ever thought about graphs" | "I do want to second what [was] said about data type and differentiating between categorical and continuous. I think data is a little more nuanced than that. And I think that that is deceptively difficult for students to do…because a lot of numbered data can be categorical." |
| FG7 | Statistics Education | "You've done a really nice job of breaking it down into a lot of components. That would be good for a biology instructor to know these are the important things to be looking for" | "I would want the graph interpretation section to maybe be fleshed out a little bit more, with more things to look for at. That made me think maybe graph structure 3C you said 'follow conventions' and maybe colors should be added, too. There are some conventions out there now with color." |
| FG8 | Statistics/Data Science Education | "Seems to do a pretty good job of capturing elements of creating the appropriate graphs and interpreting graphs and laying it out pretty nicely…I think this is a really good idea for a framework." | "In Data Form, or a couple other places…the difference between sample and aggregate data. Again, I'm used to thinking in terms of the distinction between samples and populations. Now I'm not sure if aggregate here refers to population, contracts or sample refers to some statistical functions of aggregation of the data like a mean, as opposed to each individual data point for someone entirely." |

| Table C. Alignment of GCCM Activities with 149 papers from scoping literature review. Full citations for the articles listed in Table D. | | |
| --- | --- | --- |
| GCCM Component | GCCM Activity | Scoping Review References |
| Data Selection | Data Type | 2, 7, 8, 9, 23, 40, 49, 58, 60, 62, 72, 74, 81, 85, 90, 92, 102, 103, 123, 125, 137, 140, 141, 142 |
|  | Variable Relevance | 7, 8, 9, 13, 14, 25, 50, 57, 53, 62, 61, 64, 65, 68, 72, 92, 95, 104, 107, 119, 131, 140, 142 |
|  | Variable Categorization | 1, 2, 7, 8, 9, 12, 13, 22, 23, 24, 25, 29, 30, 50, 52, 60, 62, 63, 65, 68, 74, 95, 98, 102, 107, 108, 110, 113, 119, 124, 125, 126, 138, 139, 140, 141, 142, 147 |
|  | Data Filtering/Prioritization | 30, 35, 51, 61, 68, 89, 101, 110, 114, 142 |
| Data Exploration | Data Form | 7, 8, 9, 24, 25, 29, 35, 76, 81, 85, 95, 107, 114, 116, 117, 123, 124, 140, 142 |
|  | Data Summarization | 7, 8, 9, 13, 16, 25, 35, 58, 73, 76, 81, 85, 101, 102, 107, 112, 114, 123, 137, 140, 142, 147 |
|  | Statistics Selection | 8, 9, 25, 29, 35, 58, 73, 76, 81, 89, 101, 110, 112, 114, 116, 117, 140, 142 |
|  | Data Variability | 7, 9, 18, 25, 35, 37, 40, 72, 73, 76, 81, 85, 89, 114, 116, 117, 134, 137, 140, 141 |
| Graph Assembly | Graph Type | 2, 5, 7, 8, 9, 11, 12, 13, 23, 25, 27, 29, 30, 37, 39, 40, 46, 49, 53, 55, 56, 57, 58, 59, 60, 61, 62, 64, 71, 72, 74, 75, 76, 81, 85, 83, 85, 90, 92, 102, 103, 104, 107, 114, 119, 123, 126, 137, 138, 140, 141, 142 |
|  | Data Plotting | 1, 2, 3, 7, 8, 9, 10, 11, 12, 13, 15, 16, 19, 20, 21, 25, 29, 31, 34, 35, 39, 42, 45, 46, 47, 48, 51, 52, 55, 56, 58, 59, 60, 62, 63, 65, 66, 67, 68, 71, 72, 75, 78, 81, 84, 85, 86, 87, 89, 90, 91, 92, 93, 96, 99, 100, 101, 102, 103, 104, 107, 111, 113, 114, 129, 130, 133, 134, 136, 139, 142, 143, 147 |
|  | Graph Structure | 1, 2, 3, 7, 8, 9, 10, 11, 12, 13, 16, 19, 22, 27, 28, 29, 35, 39, 44, 47, 49, 51, 52, 55, 58, 59, 60, 61, 62, 63, 64, 65, 66, 67, 68, 70, 71, 72, 74, 75, 76, 78, 81, 85, 89, 90, 91, 93, 95, 96, 98, 101, 102, 103, 104, 107, 112, 113, 114, 116, 117, 120, 127, 128, 129, 130, 133, 137, 139, 142, 143, 147 |
|  | Graph Labeling | 2, 3, 7, 8, 9, 10, 11, 13, 19, 21, 23, 24, 25, 29, 31, 39, 43, 47, 49, 51, 55, 56, 58, 59, 60, 61, 62, 63, 64, 65, 66, 68, 72, 75, 76, 81, 84, 85, 88, 89, 90, 91, 93, 95, 101, 102, 103, 104, 107, 110, 112, 113, 114, 116, 117, 119, 127, 128, 129, 130, 133, 135, 137, 142, 143 |
|  | Graph Communication | 3, 7, 8, 9, 16, 21, 25, 27, 28, 29, 31, 40, 43, 44, 47, 49, 51, 54, 55, 56, 58, 59, 60, 61, 65, 72, 75, 76, 78, 80, 81, 82, 83, 84, 85, 89, 90, 92, 102, 107, 111, 113, 114, 116, 117, 118, 119, 123, 127, 130, 137, 140, 142, 143, 146 |
| Graph Reflection | Data Points | 2, 3, 4, 5, 6, 9, 10, 12, 13, 14, 15, 16, 17, 18, 19, 20, 21, 22, 23, 24, 26, 27, 28, 29, 30, 31, 32, 33, 34, 35, 38, 39, 40, 41, 43, 46, 48, 49, 50, 51, 52, 53, 54, 56, 57, 59, 61, 63, 64, 65, 66, 67, 68, 70, 71, 73, 75, 76, 78, 81, 82, 83, 84, 85, 86, 88, 90, 93, 94, 95, 96, 97, 98, 99, 100, 101, 105, 106, 107, 108, 110, 111, 113, 114, 115, 118, 120, 121, 122, 126, 127, 128, 131, 133, 134, 135, 136, 137, 138, 141, 142, 143, 144, 145, 146, 148, 149 |
|  | Data Description | 2, 3, 4, 5, 6, 7, 9, 10, 11, 12, 13, 14, 15, 16, 17, 18, 19, 21, 22, 23, 24, 26, 27, 28, 29, 30, 31, 32, 33, 34, 35, 36, 38, 39, 40, 41, 42, 43, 44, 46, 48, 49, 50, 51, 52, 53, 54, 56, 57, 59, 61, 63, 64, 65, 66, 67, 68, 69, 70, 72, 73, 75, 76, 77, 78, 79, 80, 81, 82, 83, 84, 86, 88, 89, 91, 92, 93, 94, 95, 96, 97, 98, 99, 100, 101, 102, 104, 105, 106, 107, 108, 110, 111, 113, 114, 115, 116, 117, 118, 120, 121, 122, 124, 125, 126, 127, 128, 131, 132, 133, 134, 135, 136, 137, 138, 139, 141, 142, 143, 144, 145, 146, 147, 148, 149 |
|  | Graph Selection | 5, 7, 9, 22, 27, 30, 43, 46, 49, 51, 54, 55, 56, 59, 70, 72, 75, 76, 78, 80, 81, 82, 83, 85, 90, 92, 114, 119, 137, 140 |
|  | Scientific Claim | 2, 4, 6, 7, 9, 10, 11, 12, 13, 14, 15, 16, 17, 18, 19, 21, 22, 23, 24, 25, 26, 28, 29, 30, 31, 32, 33, 34, 35, 38, 39, 41, 43, 44, 46, 49, 50, 51, 52, 53, 54, 55, 56, 57, 59, 61, 62, 63, 64, 65, 66, 67, 68, 69, 70, 72, 75, 76, 77, 78, 81, 82, 83, 86, 89, 92, 93, 94, 95, 96, 97, 98, 99, 100, 101, 102, 104, 105, 106, 107, 108, 110, 111, 114, 115, 116, 117, 118, 119, 120, 124, 125, 126, 127, 128, 131, 132, 133, 134, 135, 136, 138, 141, 142, 145, 146, 148, 149 |

| Table D. Scoping Review References | |
| --- | --- |
| Reference Number | Full Citation |
| 1 | Åberg-Bengtsson, L. (2006). “Then you can take half … almost” — Elementary students learning bar graphs and pie charts in a computer-based context. *The Journal of Mathematical Behavior*, *25*(2), 116–135. https://doi.org/10.1016/J.JMATHB.2006.02.007 |
| 2 | Aksoy, B., & Namal, R. (2019). Scope Validity of the Graph Drawing and Interpretation Skill Checklist. *International Education Studies*, *13*(1), 76. https://doi.org/10.5539/ies.v13n1p76 |
| 3 | Alverson, C. Y., Yamamoto, S. H., Alverson, C. Y., & Yamamoto, S. H. (2016). Educational Decision Making With Visual Data and Graphical Interpretation. *SAGE Open*, *6*(4). https://doi.org/10.1177/2158244016678290 |
| 4 | Amin, B. D., Sahib, E. P., Harianto, Y. I., Patandean, A. J., Herman, & Sujiono, E. H. (2020). The interpreting ability on science kinematics graphs of senior high school students in South Sulawesi, Indonesia. *Jurnal Pendidikan IPA Indonesia*, *9*(2), 179–186. https://doi.org/10.15294/jpii.v9i2.23349 |
| 5 | Anastopoulou, S., Sharples, M., & Baber, C. (2011). An evaluation of multimodal interactions with technology while learning science concepts. *British Journal of Educational Technology*, *42*(2), 266–290. https://doi.org/10.1111/J.1467-8535.2009.01017.X |
| 6 | Angra, A., & Gardner, S. M. (2016). Development of a framework for graph choice and construction. *Advances in Physiology Education*, *40*(1), 123–128. https://doi.org/10.1152/ADVAN.00152.2015/SUPPL_FILE/SUPPLEMENTAL_WORKSHEET.PDF |
| 7 | Angra, A., & Gardner, S. M. (2017). Reflecting on Graphs: Attributes of Graph Choice and Construction Practices in Biology. *CBE Life Sciences Education*, *16*(3). https://doi.org/10.1187/CBE.16-08-0245 |
| 8 | Angra, A., & Gardner, S. M. (2018). The graph rubric: Development of a teaching, learning, and research tool. *CBE Life Sciences Education*, *17*(4). <https://doi.org/10.1187/cbe.18-01-0007> |
| 9 | Aoyama, K., & Stephens, M. (2003). Graph interpretation aspects of statistical literacy: A Japanese perspective. *Mathematics Education Research Journal*, *15*(3), 207–225. https://doi.org/10.1007/BF03217380 |
| 10 | Arteaga, P., Díaz-Levicoy, D., & Batanero, C. (2020). Chilean primary school children’s understanding of statistical graphs. *Acta Scientiae*, *22*(5), 2–24. https://doi.org/10.17648/acta.scientiae.5884 |
| 11 | Atherton, R. (2006). Grappling with climate graphs. In *Source: Teaching Geography* (Vol. 31, Issue 2). |
| 12 | Atkins, R. M., & Mcneal, K. S. (2018). Exploring Differences Among Student Populations During Climate Graph Reading Tasks: An Eye Tracking Study. In *Journal of Astronomy & Earth Sciences Education-December* (Vol. 5, Issue 2). |
| 13 | Bahtaji, M. A. A. (2020). Improving students graphing skills and conceptual understanding using explicit graphical physics instructions. *Cypriot Journal of Educational Sciences*, *15*(4), 843–853. https://doi.org/10.18844/cjes.v15i4.5063 |
| 14 | Barker, S., & Beare, R. (1999). Teaching population strategies: an evaluation of approaches. *Journal of Biological Education*, *33*(3), 149–154. https://doi.org/10.1080/00219266.1999.9655655 |
| 15 | Barnes, H. (2000). Plotting for success - or running away with graphs. *Physics Education*, *35*(5), 339. https://doi.org/10.1088/0031-9120/35/5/304 |
| 16 | Barton, E. E., & Reichow, B. (2012). Guidelines for Graphing Data With Microsoft® Office 2007^TM^, Office 2010^TM^, and Office for Mac^TM^ 2008 and 2011. Journal of Early Intervention, 34(3), 129-150. https://doi.org/10.1177/1053815112456601 |
| 17 | Beckmann, C. E., Laing, R. A., Channell, D. E., & Greenwood, J. J. (2020). Activities: Interpreting Graphs. *The Mathematics Teacher*, 82(5), 353-360. https://doi.org/10.5951/mt.82.5.0353 |
| 18 | Bell, A., & Janvier, C. (1981). The Interpretation of Graphs Representing Situations on JSTOR. *For the Learning of Mathematics*, *2*(1), 34–42. https://www.jstor.org/stable/40240746 |
| 19 | Berg, C. A., & Phillips, D. G. (1994). An investigation of the relationship between logical thinking structures and the ability to construct and interpret line graphs. *Journal of Research in Science Teaching*, *31*(4), 323–344. https://doi.org/10.1002/TEA.3660310404 |
| 20 | Berg, C., & Boote, S. (2017). Format Effects of Empirically Derived Multiple-Choice Versus Free-Response Instruments When Assessing Graphing Abilities. *International Journal of Science and Mathematics Education*, *15*(1), 19–38. https://doi.org/10.1007/S10763-015-9678-6/FIGURES/5 |
| 21 | Bestgen, B. J. (1980). Making and Interpreting Graphs and Tables: Results and Implications from National Assessment. *The Arithmetic Teacher*, *28*(4), 26–29. https://doi.org/10.5951/AT.28.4.0026 |
| 22 | Boote, S. K. (2014). Assessing and Understanding Line Graph Interpretations Using a Scoring Rubric of Organized Cited Factors. *Journal of Science Teacher Education*, *25*(3), 333–354. https://doi.org/10.1007/S10972-012-9318-8/TABLES/2 |
| 23 | Boote, S. K., & Boote, D. N. (2017). Leaping from Discrete to Continuous Independent Variables: Sixth Graders’ Science Line Graph Interpretations. *Https://Doi.Org/10.1086/690204*, *117*(3), 455–484. https://doi.org/10.1086/690204 |
| 24 | Bowen, G. M., & Roth, W. M. (2003). Graph interpretation practices of science and education majors. *Canadian Journal of Science, Mathematics and Technology Education*, *3*(4), 499–512. https://doi.org/10.1080/14926150309556585/METRICS |
| 25 | Bowen, G. M., & Roth, W. M. (2005). Data and graph interpretation practices among preservice science teachers. *Journal of Research in Science Teaching*, *42*(10), 1063–1088. https://doi.org/10.1002/TEA.20086 |
| 26 | Bowen, G. M., Roth, W. M., & McGinn, M. K. (1999). Interpretations of graphs by university biology students and practicing scientists: Toward a social practice view of scientific representation practices. *Journal of Research in Science Teaching*, *26*, 1020–1043. https://onlinelibrary.wiley.com/doi/epdf/10.1002/%28SICI%291098-2736%28199911%2936%3A9%3C1020%3A%3AAID-TEA4%3E3.0.CO%3B2-%23 |
| 27 | Bradstreet, T. E., & Palcza, J. S. (2012). Digging into data with graphics. *Teaching Statistics*, *34*(2), 68–74. https://doi.org/10.1111/J.1467-9639.2011.00490.X |
| 28 | Bragdon, D., Pandiscio, E., & Speer, N. (2019). University students’ graph interpretation and comprehension abilities. *Investigations in Mathematics Learning*, *11*(4), 275–290. https://doi.org/10.1080/19477503.2018.1480862 |
| 29 | Brasell, H. M., & Rowe, M. B. (1993). Graphing Skills Among High School Physics Students. *School Science and Mathematics*, *93*(2), 63–70. https://doi.org/10.1111/J.1949-8594.1993.TB12196.X |
| 30 | Brugar, K. A. (2017). “We don’t have students colour maps anymore …” a survey of social studies teachers use of visual materials. *Journal of Visual Literacy*, *36*(3–4), 142–163. https://doi.org/10.1080/1051144X.2017.1397380 |
| 31 | Bursal, M., & Yetiş, S. (2020). Middle School Students’ Graph Skills and Affective States about Graphs. *International Journal of Research in Education and Science (IJRES)*, *6*(4), 692–704. www.ijres.net |
| 32 | Burson, G. (1990). Using Charts and Graphs to Teach Immigration History. *OAH Magazine of History*, *4*(4), 46–49. https://www.jstor.org/stable/25162697 |
| 33 | Bykerk-Kauffman, A. (2008). The Moon Project: Student Research and Lesson Design in an Introductory Geoscience Course for Pre-service Teachers. *Journal of Geoscience Education*, *56*(5), 434–439. https://doi.org/10.5408/JGE_NOV2008_BYKERK_377 |
| 34 | Callingham, R., & Watson, J. M. (2005). Measuring statistical literacy. *Journal of Applied Measurement*, *6*(1), 19–47. https://europepmc.org/article/med/15701942 |
| 35 | Çatman Aksoy, E., & Işıksal Bostan, M. (2021). Seventh Graders’ Statistical Literacy: an Investigation on Bar and Line Graphs. *International Journal of Science and Mathematics Education*, *19*(2), 397–418. https://doi.org/10.1007/s10763-020-10052-2 |
| 36 | Cave, R. C. (1995). Graphing, bit by bit. *The Mathematics Teacher*, *88*(5), 372–372. https://www.proquest.com/openview/5e1c61dd30bc96ed9ee340393642f0bd/1?pq-origsite=gscholar&cbl=41299 |
| 37 | Çelik, H., & Pektaş, H. M. (2017). Graphic Comprehension and Interpretation Skills of Preservice Teachers with Different Learning Approaches in a Technology-Aided Learning Environment. *International Journal of Science and Mathematics Education*, *15*(1), 1–17. https://doi.org/10.1007/S10763-015-9667-9 |
| 38 | Christner, L., & Kleier, C. (2011). Quantitative reasoning in introductory environmental science textbooks. *Journal of Environmental Studies and Sciences*, *1*(4), 296–300. https://doi.org/10.1007/S13412-011-0063-7/FIGURES/1 |
| 39 | Çil, E., & Kar, H. (2015). Pre-Service Science Teachers’ Interpretations of Graphs: A Cross-Sectional Study. *Science Educator*, *24*(1), 36–44. |
| 40 | Clary, R., & Wandersee, J. (2014). Graphing the Past. *The Science Teacher*, *081*(05). https://doi.org/10.2505/4/TST14_081_05_39 |
| 41 | Cantoral, R., Moreno‑Durazo, A., & Caballero‑Pérez, M. (2018). Socio-epistemological research on mathematical modelling: An empirical approach to teaching and learning. *ZDM - Mathematics Education*, 50(1–2), 77–89. https://doi.org/10.1007/s11858-018-0922-8 |
| 42 | Cooper, L. L., & Shore, F. S. (2010). The Effects of Data and Graph Type on Concepts and Visualizations of Variability. *Journal of Statistics Education*, *18*(2), 1–16. https://doi.org/10.1080/10691898.2010.11889487 |
| 43 | Cortés-Figueroa, J. E., Moore-Russo, D. A., & Case, M. (2004). Promoting Graphical Thinking: Using Temperature and a Graphing Calculator To Teach Kinetics Concepts. *Journal of Chemical Education*, *81*(1), 69–71. https://doi.org/10.1021/ED081P69 |
| 44 | D’Eon, J. C., Stirchak, L. T., Brown, A. S., & Saifuddin, Y. (2021). Project-Based Learning Experience That Uses Portable Air Sensors to Characterize Indoor and Outdoor Air Quality. *Journal of Chemical Education*, 98(2). https://doi.org/10.1021/acs.jchemed.0c00222 |
| 45 | Dart, E. H., & Radley, K. C. (2018). Toward a standard assembly of linear graphs. *School Psychology Quarterly*, *33*(3), 350–355. https://doi.org/10.1037/spq0000269 |
| 46 | Rycker, T. de. (2001). Analyzing tables, graphs, and charts: A four-step approach. (My favorite assignment). *Business Communication Quarterly*, 64(4), 72–83. https://go.gale.com/ps/i.do?p=AONE&sw=w&issn=10805699&v=2.1&it=r&id=GALE%7CA80950644&sid=googleScholar&linkaccess=fulltext |
| 47 | Delgado, C., & Lucero, M. M. (2015). Scale construction for graphing: An investigation of students’ resources. *Journal of Research in Science Teaching*, *52*(5), 633–658. https://doi.org/10.1002/TEA.21205 |
| 48 | Dixon, J. K., & Falba, C. J. (1997). Graphing in the Information Age: Using Data from the World Wide Web. *Mathematics Teaching in the Middle School*, *2*(5), 298–304. |
| 49 | Dixon, M. R., Jackson, J. W., Small, S. L., Horner‐King, M. J., Lik, N. M. K., Garcia, Y., & Rosales, R. (2009). CREATING SINGLE-SUBJECT DESIGN GRAPHS IN MICROSOFT EXCELTM 2007. *Journal of Applied Behavior Analysis*, *42*(2), 277. https://doi.org/10.1901/JABA.2009.42-277 |
| 50 | Doerr, H. M., & Zangor, R. (2000). Creating meaning for and with the graphing calculator. *Educational Studies in Mathematics*, *41*(2), 143–163. https://doi.org/10.1023/A:1003905929557/METRICS |
| 51 | Donnelly-Hermosillo, D. F., Gerard, L. F., & Linn, M. C. (2020). Impact of graph technologies in K-12 science and mathematics education. *Computers and Education*, *146*. https://doi.org/10.1016/j.compedu.2019.103748 |
| 52 | Duesbery, L., Braun-Monegan, J., Liu, K., & McCoy, J. (2017). Thinking critically about data displays. *Journal of Visual Literacy*, *36*(1), 41–54. https://doi.org/10.1080/1051144X.2017.1331681 |
| 53 | Duijzer, C., Van den Heuvel-Panhuizen, M., Veldhuis, M., & Doorman, M. (2019). Supporting primary school students’ reasoning about motion graphs through physical experiences. *ZDM - Mathematics Education*, *51*(6), 899–913. https://doi.org/10.1007/s11858-019-01072-6 |
| 54 | Duplass, J. A. (1996). Charts, Tables, Graphs, and Diagrams: An Approach for Social Studies Teachers. *The Social Studies*, *87*(1), 32–38. https://doi.org/10.1080/00377996.1996.10114492 |
| 55 | Engebretsen, M. (2020). From Decoding a Graph to Processing a Multimodal Message Interacting with data visualisation in the news media. *Nordicom Review*, *41*(1), 33–50. https://doi.org/10.1021/acs.jchemed.0c00222 |
| 56 | Enzingmüller, C., & Prechtl, H. (2021). Constructing Graphs in Biology Class: Secondary Biology Teachers’ Beliefs, Motivation, and Self-Reported Practices. *International Journal of Science and Mathematics Education*, *19*(1), 1–19. https://doi.org/10.1007/s10763-019-09975-2 |
| 57 | Ergül, N. R. (2018). Pre-Service Science Teachers’ Construction and Interpretation of Graphs. *Universal Journal of Educational Research*, *6*(1), 139–144. https://doi.org/10.13189/ujer.2018.060113 |
| 58 | Fernandes, J. A., & Freitas, A. (2019). Selection and Application of graphical and numerical statistical tools by prospective primary school teachers. *Acta Scientiae*, *21*(6), 82–97. https://doi.org/10.17648/acta.scientiae.5344 |
| 59 | Fitzallen, N., Watson, J., & Wright, S. (2017). The Heat Is On! Using a Stylised Graph to Engender Understanding. *Australian Primary Mathematics Classroom*, *22*(2), 3–7. |
| 60 | Garcia-Mila, M., Marti, E., Gilabert, S., & Castells, M. (2014). Fifth Through Eighth Grade Students’ Difficulties in Constructing Bar Graphs: Data Organization, Data Aggregation, and Integration of a Second Variable. *Mathematical Thinking and Learning*, *16*(3), 201–233. https://doi.org/10.1080/10986065.2014.921132 |
| 61 | Glazer, N. (2011). Challenges with graph interpretation: a review of the literature. *Studies in Science Education*, *47*(2), 183–210. https://doi.org/10.1080/03057267.2011.605307 |
| 62 | Gültepe, N. (2016). Reflections on high school students’ graphing skills and their conceptual understanding of drawing chemistry graphs. *Educational Sciences Theory & Practice*, *16*(1), 53–81. https://doi.org/10.12738/ESTP.2016.1.2837 |
| 63 | Harris, D., & Zwiep, S. G. (2013). BEYOND SLOPES and POINTS: Teaching students how graphs describe the relationships between scientific phenomena. *The Science Teacher*, 80(3), 43–47. http://www.jstor.org/stable/43557707 |
| 64 | Harsh, J. A., Campillo, M., Murray, C., Myers, C., Nguyen, J., & Maltese, A. V. (2019). Seeing data like an expert: An eye-tracking study using graphical data representations. *CBE Life Sciences Education*, *18*(3). https://doi.org/10.1187/cbe.18-06-0102 |
| 65 | Highfill, J. K., & Weber, W. V. (1990). Graphing to Learn Economics. *The Social Studies*, *81*(2), 53–58. https://doi.org/10.1080/00377996.1990.9957494 |
| 66 | Hoffmann, M. H. G., & Roth, W. M. (2005). What you should know to survive in knowledge societies: On a semiotic understanding of ‘knowledge.’ *Semiotica*, *157*, 105–142. https://doi.org/10.1515/SEMI.2005.2005.157.1-4.105 |
| 67 | Ingulfsen, L., Furberg, A., & Strømme, T. A. (2018). Students’ engagement with real-time graphs in CSCL settings: scrutinizing the role of teacher support. *International Journal of Computer-Supported Collaborative Learning*, *13*(4), 365–390. https://doi.org/10.1007/s11412-018-9290-1 |
| 68 | Kastberg, S. E., D’Ambrosio, B. S., Lynch-Davis, K., Mintos, A., & Krawczyk, K. (2013). CCSSM Challenge: Graphing Ratio and Proportion. *Mathematics Teaching in the Middle School*, *19*(5), 294–300. |
| 69 | Knöchelmann, N., Krueger, S., Flack, A., & Osterhaus, C. (2019). Adults’ ability to interpret covariation data presented in bar graphs depends on the context of the problem. *Frontline Learning Research*, *7*(4), 58–65. https://doi.org/10.14786/flr.v7i4.471 |
| 70 | Kontogianni, A., & Tatsis, K. (2018). Investigating adults’ statistical literacy in a Second Chance School through the teaching of graphs. In *Adults Learning Mathematics: An International Journal* (Vol. 13, Issue 1). |
| 71 | Kramarski, B. (1999). The Study Of Graphs By Computers: Is Easier Better? *Education Media International*, *21*(1), 203–209. https://doi.org/10.1080/0952398990360306 |
| 72 | Kubina, R. M., Kostewicz, D. E., Brennan, K. M., & King, S. A. (2017). A Critical Review of Line Graphs in Behavior Analytic Journals. *Educational Psychology Review*, *29*(3), 583–598. https://doi.org/10.1007/S10648-015-9339-X |
| 73 | Kukliansky, I., Kukliansky, & Ida. (2016). Student’s Conceptions in Statistical Graph’s Interpretation. *International Journal of Higher Education*, *5*(4). https://EconPapers.repec.org/RePEc:jfr:ijhe11:v:5:y:2016:i:4:p:262 |
| 74 | LaDue, N. D., Libarkin, J. C., & Thomas, S. R. (2015). Visual representations on high school biology, chemistry, earth science, and physics assessments. *Journal of Science Education and Technology*, *24*(6), 818–834. https://doi.org/10.1007/S10956-015-9566-4/FIGURES/11 |
| 75 | Lai, Kevin; Cabrera, Julio; Vitale, Jonathan M.; Madhok, Jacquie; Tinker, Robert; Linn, Marcia C. |
| 76 | Lane, D. M., & Sándor, A. (2009). Designing better graphs by including distributional information and integrating words, numbers, and images. *Psychological Methods*, *14*(3), 239–257. https://doi.org/10.1037/A0016620 |
| 77 | Lane, J. D., Shepley, C., & Spriggs, A. D. (2021). Issues and Improvements in the Visual Analysis of A-B Single-Case Graphs by Pre-Service Professionals. *Remedial and Special Education*, *42*(4), 235–247. https://doi.org/10.1177/0741932519873120 |
| 78 | Larson, M. J., & Whitin, D. J. (2010). Young Children Use Graphs to Build Mathematical Reasoning. *Dimensions of Early Childhood*, *38*(3), 15–22. |
| 79 | Lem, S., Onghena, P., Verschaffel, L., & Van Dooren, W. (2013). On the misinterpretation of histograms and box plots. *Educational Psychology*, *33*(2), 155–174. https://doi.org/10.1080/01443410.2012.674006 |
| 80 | Lem, S., Onghena, P., Verschaffel, L., & Van Dooren, W. (2017). The power of refutational text: changing intuitions about the interpretation of box plots. *European Journal of Psychology of Education*, *32*(4), 537–550. https://doi.org/10.1007/S10212-016-0320-Y/TABLES/3 |
| 81 | Manisera, M. (2011). A graphical tool to compare groups of subjects on categorical variables. *Electronic Journal of Applied Statistical Analysis*, *4*(1), 1–22. https://doi.org/10.1285/I20705948V4N1P1 |
| 82 | Marzocchi, A. S., Turner, K., & Druken, B. K. (2019). Using Graph Talks to Engage Undergraduates in Conversations Around Social Justice. *PRIMUS*, *29*(3–4), 375–395. https://doi.org/10.1080/10511970.2018.1456499 |
| 83 | Matuk, C., Zhang, J., Uk, I., & Linn, M. C. (2019). Qualitative graphing in an authentic inquiry context: How construction and critique help middle school students to reason about cancer. *Journal of Research in Science Teaching*, *56*(7), 905–936. https://doi.org/10.1002/tea.21533 |
| 84 | McMillen, S., & McMillen, B. (2010). My Bar Graph Tells a Story. *Teaching Children Mathematics*, *16*(7), 430–436. |
| 85 | Mhlolo, M. (2015). Investigating Learners’ Meta-Representational Competencies When Constructing Bar Graphs. *Pythagoras*, *36*(1). |
| 86 | Mitnik, R., Recabarren, M., Nussbaum, M., & Soto, A. (2009). Collaborative robotic instruction: A graph teaching experience. *Computers & Education*, *53*(2), 330–342. https://doi.org/10.1016/J.COMPEDU.2009.02.010 |
| 87 | Moody, M. (1990). Activities. Mathematics as Communication: Graphing Information Collected Over Time. *Mathematics Teacher*, *83*(9), 730–736. |
| 88 | Moreno-Esteva, E. G., White, S. L. J., Wood, J. M., & Black, A. A. (2018). Application of mathematical and machine learning techniques to analyse eye tracking data enabling better understanding of children’s visual cognitive behaviours. *Frontline Learning Research*, 6(3), 72–84. https://doi.org/10.14786/flr.v6i3.365 |
| 89 | Newell, K. W., & Christ, T. J. (2017). Novice Interpretations of Progress Monitoring Graphs: Extreme Values and Graphical Aids. *Http://Dx.Doi.Org/10.1177/1534508417694855*, *42*(4), 224–236. https://doi.org/10.1177/1534508417694855 |
| 90 | Nibbelink, W. (1982). Graphing for Any Grade. *The Arithmetic Teacher*, *30*(3), 28–31. https://doi.org/10.5951/AT.30.3.0028 |
| 91 | Nivens, R. A. (2016). Using graphing to reveal the hidden transformations in palindrome (and other types of) licence plates \| The Australian Mathematics Teacher. *The Australian Mathematics Teacher*, *72*(2), 33–38. https://search.informit.org/doi/10.3316/informit.194358967419881 |
| 92 | Parker, J., & Carroll Widmer, C. (2020). Teaching Mathematics with Technology: Statistics and Graphing. *The Arithmetic Teacher*, *39*(8), 48–52. https://doi.org/10.5951/AT.39.8.0048 |
| 93 | Parmar, R. S., & Signer, B. R. (2005). Sources of error in constructing and interpreting graphs: A study of fourth- and fifth-grade students with LD. *Journal of Learning Disabilities*, *38*(3), 250–261. https://doi.org/10.1177/00222194050380030601 |
| 94 | Patahuddin, S. M., & Lowrie, T. (2019). Examining Teachers’ Knowledge of Line Graph Task: a Case of Travel Task. *International Journal of Science and Mathematics Education*, *17*(4), 781–800. https://doi.org/10.1007/s10763-018-9893-z |
| 95 | Pechenik, J. A., & Tashiro, J. S. (1992). The Graphing Detective: An Exercise in Critical Reading, Experimental Design and Data Analysis. *American Biology Teacher*, *54*(7), 432–435. |
| 96 | Pfannkuch, M., Regan, M., Wild, C., & Horton, N. J. (2010). Telling Data Stories: Essential Dialogues for Comparative Reasoning. *Journal of Statistics Education*, *18*(1), 1–38. https://doi.org/10.1080/10691898.2010.11889479 |
| 97 | Phage, I. B., Lemmer, M., & Hitge, M. (2017). Probing factors influencing students’ graph comprehension regarding four operations in kinematics graphs. *African Journal of Research in Mathematics, Science and Technology Education*, *21*(2), 200–210. https://doi.org/10.1080/18117295.2017.1333751 |
| 98 | Pierce, R., & Chick, H. (2013). Workplace statistical literacy for teachers: Interpreting box plots. *Mathematics Education Research Journal*, *25*(2), 189–205. https://doi.org/10.1007/S13394-012-0046-3/TABLES/5 |
| 99 | Piston, C. (1992). Supplementing the Graphing Curriculum. *Mathematics Teacher*, *85*(5), 336–341. |
| 100 | Pitts Bannister, Vanessa R; Jamar, Idorenyin; Mutegi, Jomo W.  (2007). *Science and Children,* 45(2), 30-32. |
| 101 | Pols, C. F. J., Dekkers, P. J. J. M., & de Vries, M. J. (2021). What do they know? Investigating students’ ability to analyse experimental data in secondary physics education. *International Journal of Science Education*, *43*(2), 274–297. https://doi.org/10.1080/09500693.2020.1865588 |
| 102 | Preece, J., & Janvier, C. (1992). A Study of the Interpretation of Trends in Multiple Curve Graphs of Ecological Situations. *School Science and Mathematics*, *92*(6), 299–306. |
| 103 | Rezba, R. J., Giese, R. N., & Cothron, J. H. (1998). Graphing Is a Snap. *Science Scope*, *21*(4), 20–23. http://www.jstor.org/stable/43177144 |
| 104 | Rodriguez, J. M. G., Bain, K., Towns, M. H., Elmgren, M., & Ho, F. M. (2019). Covariational reasoning and mathematical narratives: Investigating students’ understanding of graphs in chemical kinetics. *Chemistry Education Research and Practice*, *20*(1), 107–119. https://doi.org/10.1039/c8rp00156a |
| 105 | Rodriguez, J. M. G., Harrison, A. R., & Becker, N. M. (2020). Analyzing Students’ Construction of Graphical Models: How Does Reaction Rate Change over Time? *Journal of Chemical Education*, *97*(11), 3948–3956. https://doi.org/10.1021/acs.jchemed.0c01036 |
| 106 | Roth, W. M. (2002). Reading graphs: Contributions to an integrative concept of literacy. *Journal of Curriculum Studies*, *34*(1), 1–24. https://doi.org/10.1080/00220270110068885 |
| 107 | Roth, W.-M. (2005). Mathematical Inscriptions and the Reflexive Elaboration of Understanding: An Ethnography of Graphing and Numeracy in a Fish Hatchery. *Mathematical Thinking and Learning*, *7*(2), 75–110. https://doi.org/10.1207/S15327833MTL0702_1 |
| 108 | Roth, W. M., & Hwang, S. W. (2006). On the relation of abstract and concrete in scientists’ graph interpretations: A case study. *The Journal of Mathematical Behavior*, *25*(4), 318–333. https://doi.org/10.1016/J.JMATHB.2006.11.005 |
| 109 | Roth, W. M., & Lee, Y. J. (2004). Interpreting unfamiliar graphs: A generative, activity theoretic model. *Educational Studies in Mathematics*, *57*(2), 265–290. https://doi.org/10.1023/B:EDUC.0000049276.37088.E4 |
| 110 | Roth, W.-M., & Bowen, G. M. (1999). Of Cannibals, Missionaries, and Converts: Graphing Competencies from Grade 8 to Professional Science Inside (Classrooms) and Outside (Field/Laboratory). *Science, Technology, and Human Values*, *24*(2), 179–212. |
| 111 | Roth, W. M., & Bowen, G. M. (2003). When Are Graphs Worth Ten Thousand Words? An Expert-Expert Study. *Cognition and Instruction*, *21*(4), 429–473. https://doi.org/10.1207/S1532690XCI2104_3 |
| 112 | Roth, W.-M., Bowen, G. M., & McGinn, M. K. (1999). Differences in Graph-Related Practices between High School Biology Textbooks and Scientific Ecology Journals. *Journal of Research in Science Teaching*, *36*(9), 977–1019. |
| 113 | Roth, W. M., & Maheux, J. F. (2015). The stakes of movement: A dynamic approach to mathematical thinking. *Curriculum Inquiry*, *45*(3), 266–284. https://doi.org/10.1080/03626784.2015.1031629 |
| 114 | Roth, W. M., & Temple, S. (2014). On understanding variability in data: A study of graph interpretation in an advanced experimental biology laboratory. *Educational Studies in Mathematics*, *86*(3), 359–376. https://doi.org/10.1007/S10649-014-9535-5 |
| 115 | Rule, A. C., & Meyer, M. A. (2009). Teaching Urban High School Students Global Climate Change Information and Graph Interpretation Skills Using Evidence from the Scientific Literature. *Journal of Geoscience Education*, *57*(5), 335–347. https://doi.org/10.5408/1.3559674 |
| 116 | Sakshaug, Lynae E.; Wohlhuter, Kay A. |
| 117 | Saterbak, A., Moturu, A., & Volz, T. (2018). Using a Teaching Intervention and Calibrated Peer Review^TM^ Diagnostics to Improve Visual Communication Skills. *Annals of Biomedical Engineering*, *46*(3), 513–524. https://doi.org/10.1007/S10439-017-1946-X/FIGURES/3 |
| 118 | Şefik, Ö., & Dost, Ş. (2020). The analysis of the understanding of the three-dimensional (Euclidian) space and the two-variable function concept by university students. *Journal of Mathematical Behavior*, *57*. https://doi.org/10.1016/j.jmathb.2019.03.004 |
| 119 | Shand, K. (2009). The Interplay of Graph and Text in the Acquisition of Historical Constructs. *Theory and Research in Social Education*, *37*(3), 300–324. |
| 120 | Shreiner, T. L., & Dykes, B. M. (2021). Visualizing the teaching of data visualizations in social studies: A study of teachers’ data literacy practices, beliefs, and knowledge. *Theory and Research in Social Education*, *49*(2), 262–306. https://doi.org/10.1080/00933104.2020.1850382 |
| 121 | Skoumios, M. (2013). Pupils’ ability to interpret bar graphs. *International Journal of Science, Mathematics and Technology Learning*, *19*(3), 57–70. https://doi.org/10.18848/2327-7971/CGP/V19I03/49004 |
| 122 | Skrabankova, J., Popelka, S., & Beitlova, M. (2020). Students’ ability to work with graphs in physics studies related to three typical student groups. *Journal of Baltic Science Education*, *19*(2), 298–316. https://doi.org/10.33225/jbse/20.19.298 |
| 123 | So, W. W. mui. (2013). Connecting mathematics in primary science inquiry projects. *International Journal of Science and Mathematics Education*, *11*(2), 385–406. https://doi.org/10.1007/S10763-012-9342-3/METRICS |
| 124 | Song H., Park Y., Yun E. |
| 125 | Stern, E., Aprea, C., & Ebner, H. G. (2003). Improving cross-content transfer in text processing by means of active graphical representation. *Learning and Instruction*, *13*(2), 191–203. https://doi.org/10.1016/S0959-4752(02)00020-8 |
| 126 | Szyjka, S., Mumba, F., & Wise, K. (2011). CONFIRMATORY FACTOR ANALYSIS OF THE QUESTIONNAIRE OF ATTITUDE TOWARD STATISTICAL GRAPHS FOR USE IN SCIENCE EDUCATION. *Journal of Baltic Science Education*. |
| 127 | Tadayon, F., & Ravand, H. (2016). Using grounded theory to validate Bachman and Palmer’s (1996) strategic competence in EFL graph-writing. *Language Testing in Asia*, *6*(1). https://doi.org/10.1186/s40468-016-0031-y |
| 128 | Tairab, H. H., & Khalaf Al-Naqbi, A. K. (2004). How do secondary school science students interpret and construct scientific graphs? *Journal of Biological Education*, *38*(3), 127–132. https://doi.org/10.1080/00219266.2004.9655920 |
| 129 | Testa, I., Monroy, G., & Sassi, E. (2002). Students’ reading images in kinematics: The case of real-time graphs. *International Journal of Science Education*, *24*(3), 235–256. https://doi.org/10.1080/09500690110078897 |
| 130 | Tyner, B. C., & Fienup, D. M. (2015). A comparison of video modeling, text-based instruction, and no instruction for creating multiple baseline graphs in Microsoft Excel. *Journal of Applied Behavior Analysis*, *48*(3), 701–706. https://doi.org/10.1002/JABA.223 |
| 131 | Tyner, B. C., & Fienup, D. M. (2016). The Effects of Describing Antecedent Stimuli and Performance Criteria in Task Analysis Instruction for Graphing. *Journal of Behavioral Education*, *25*(3), 379–392. https://doi.org/10.1007/S10864-015-9242-Z/FIGURES/3 |
| 132 | Vaara, R. L., & Sasaki, D. G. G. (2019). Teaching kinematic graphs in an undergraduate course using an active methodology mediated by video analysis. *LUMAT*, *7*(1), 1–26. https://doi.org/10.31129/LUMAT.7.1.374 |
| 133 | van den Bosch, R. M., Espin, C. A., Pat-El, R. J., & Saab, N. (2019). Improving Teachers’ Comprehension of Curriculum-Based Measurement Progress-Monitoring Graphs. *Journal of Learning Disabilities*, *52*(5), 413–427. https://doi.org/10.1177/0022219419856013 |
| 134 | Vitale, J. M., Applebaum, L., & Linn, M. C. (2019). Coordinating between Graphs and Science Concepts: Density and Buoyancy. *Cognition and Instruction*, *37*(1), 38–72. https://doi.org/10.1080/07370008.2018.1539736 |
| 135 | Vitale, J. M., Lai, K., & Linn, M. C. (2015). Taking advantage of automated assessment of student-constructed graphs in science. *Journal of Research in Science Teaching*, *52*(10), 1426–1450. https://doi.org/10.1002/TEA.21241 |
| 136 | Volkwyn, T. S., Airey, J., Gregorcic, B., & Linder, C. (2020). Developing representational competence: linking real-world motion to physics concepts through graphs. *Learning: Research and Practice*, *6*(1), 88–107. https://doi.org/10.1080/23735082.2020.1750670 |
| 137 | Wagner, D. L., Hammerschmidt-Snidarich, S. M., Espin, C. A., Seifert, K., & McMaster, K. L. (2017). Pre-service Teachers’ Interpretation of CBM Progress Monitoring Data. *Learning Disabilities Research and Practice*, *32*(1), 22–31. https://doi.org/10.1111/LDRP.12125 |
| 138 | Wall, J. J., & Benson, C. C. (2020). So Many Graphs, So Little Time. *Mathematics Teaching in the Middle School*, *15*(2), 82–91. https://doi.org/10.5951/MTMS.15.2.0082 |
| 139 | Wang, Z. H., Wei, S., Ding, W., Chen, X., Wang, X., & Hu, K. (2012). Students’ Cognitive Reasoning of Graphs: Characteristics and progression. *International Journal of Science Education*, *34*(13), 2015–2041. https://doi.org/10.1080/09500693.2012.709333 |
| 140 | Wavering, M. J. (1989). Logical reasoning necessary to make line graphs. *Journal of Research in Science Teaching*, *26*(5), 373–379. https://doi.org/10.1002/TEA.3660260502 |
| 141 | Webber, H., Nelson, S., Weatherbee, R., Zoellick, B., & Schauffler, M. (2014). The Graph Choice Chart. *The Science Teacher*, *081*(08). https://doi.org/10.2505/4/TST14_081_08_37 |
| 142 | Whitaker, D., & Jacobbe, T. (2017). Students’ Understanding of Bar Graphs and Histograms: Results From the LOCUS Assessments. *Journal of Statistics Education*, *25*(2), 90–102. https://doi.org/10.1080/10691898.2017.1321974 |
| 143 | Wu, H. K., & Krajcik, J. S. (2006). Inscriptional practices in two inquiry-based classrooms: A case study of seventh graders’ use of data tables and graphs. *Journal of Research in Science Teaching*, *43*(1), 63–95. https://doi.org/10.1002/TEA.20092 |
| 144 | Wu, Y., & Wong, K. Y. (2007). Impact of a Spreadsheet Exploration on Secondary School Students’ Understanding of Statistical Graphs. *Journal of Computers in Mathematics and Science Teaching*, *26*(4), 355–385. |
| 145 | Xi, X. (2010). Aspects of performance on line graph description tasks: Influenced by graph familiarity and different task features. *Language Testing*, *27*(1), 73–100. https://doi.org/10.1177/0265532209346454 |
| 146 | Yang, H.-C. (2012). A comparative study of composing processes in reading- and graph-based writing tasks. *Language Testing in Asia*, *2*(3), 1–20. |
| 147 | Young, K. E., & Young, C. H. (2019). Plot Twist: Changing the Story in Graphing Skills with an Open-Ended Approach. *The Physics Teacher*, *57*(1), 44–46. https://doi.org/10.1119/1.5084928 |
| 148 | Zeuch, N., Förster, N., & Souvignier, E. (2017). Assessing Teachers’ Competencies to Read and Interpret Graphs from Learning Progress Assessment: Results from Tests and Interviews. *Learning Disabilities Research & Practice*, *32*(1), 61–70. https://doi.org/10.1111/LDRP.12126 |
| 149 | Zucker, A., Kay, R., & Staudt, C. (2014). Helping Students Make Sense of Graphs: An Experimental Trial of SmartGraphs Software. *Journal of Science Education and Technology*, *23*(3), 441–457. https://doi.org/10.1007/S10956-013-9475-3/TABLES/8 |
